# Aneuploidy and prognosis of non-small-cell lung cancer: a meta-analysis of published data

**DOI:** 10.1054/bjoc.2001.1892

**Published:** 2001-07

**Authors:** D Choma, J-P Daurès, X Quantin, J L Pujol

**Affiliations:** Thoracic Oncology Unit, Centre Hospitalier Universitaire de Montpellier, Hôpital Arnaud de Villeneuve, Montpellier Cedex, 34295, France; Department of Biostatistics and Epidemiology, University Institute for Clinical Research, Montpellier Cedex 5, Rue de la Cardonille, 34093, France

**Keywords:** ploidy, non-small-cell lung cancer, prognosis

## Abstract

In lung cancer, DNA content abnormalities have been described as a heterogeneous spectrum of impaired tumour cell DNA histogram patterns. They are merged into the common term of aneuploidy and probably reflect a high genotypic instability. In non-small-cell lung cancer, the negative effect of aneuploidy has been a subject of controversy inasmuch as studies aimed at determining the survival–DNA content relationship have reported conflicting results. We made a meta-analysis of published studies aimed at determining the prognostic effect of aneuploidy in surgically resected non-small-cell lung cancer. 35 trials have been identified in the literature. A comprehensive collection of data has been constructed taking into account the following parameters: quality of specimen, DNA content assessment method, aneuploidy definition, histology and stage grouping, quality of surgical resection and demographic characteristics of the analysed population. Among the 4033 assessable patients, 2626 suffered from non-small-cell lung cancer with aneuploid DNA content (overall frequency of aneuploidy: 0.65; 95% CI: (0.64–0.67)). The DerSimonian and Laird method was used to estimate the size effects and the Peto and Yusuf method was used in order to generate the odds ratios (OR) of reduction in risk of death for patients affected by a nearly diploid (non-aneuploid) non-small-cell lung cancer. Survivals following surgical resection, from 1 to 5 years, were chosen as the end-points of our meta-analysis. Patients suffering from a nearly diploid tumour benefited from a significant reduction in risk of death at 1, 2, 3 and 4 years with respective OR: 0.51, 0.51, 0.45 and 0.67 (*P*< 10^−4^for each end-point). 5 years after resection, the reduction of death was of lesser magnitude: OR: 0.87 (*P* = 0.08). The test for overall statistical heterogeneity was conventionally significant (*P*< 0.01) for all 5 end-points, however. None of the recorded characteristics of the studies could explain this phenomenon precluding a subset analysis. Therefore, the DerSimonian and Laird method was applied inasmuch as this method allows a correction for heterogeneity. This method demonstrated an increase in survival at 1, 2, 3, 4 and 5 years for patients with diploid tumours with respective size effects of 0.11, 0.15, 0.20, 0.20 and 0.21 (value taking into account the correction for heterogeneity;*P*< 10^−4^for each end-point). Patients who benefit from a surgical resection for non-small-cell lung cancer with aneuploid DNA content prove to have a higher risk of death. This negative prognostic factor decreases the probability of survival by 11% at one year, a negative effect deteriorating up to 21% at 5 years following surgery. © 2001 Cancer Research Campaign http://www.bjcancer.com


British Journal of Cancer (2001) 85(1), 14–22
© 2001 Cancer Research Campaign
doi: 10.1054/ bjoc.2001.1892, available online at http://www.idealibrary.com on IDEAL®

http://www.bjcancer.com

### Aneuploidy and prognosis of non-small-cell lung cancer: a meta-analysis of published data

**D Choma[1], J-P Daurès[2], X Quantin[1] and JL Pujol[1,2]**

[1]Thoracic Oncology Unit, Centre Hospitalier Universitaire de Montpellier, Hôpital Arnaud de Villeneuve, 34295 Montpellier Cedex, France; [2]Department of Biostatistics and Epidemiology, University Institute for Clinical Research, Rue de la Cardonille, 34093 Montpellier Cedex 5, France

**Summary** In lung cancer, DNA content abnormalities have been described as a heterogeneous spectrum of impaired tumour cell DNA histogram patterns. They are merged into the common term of aneuploidy and probably reflect a high genotypic instability. In non-small-cell lung cancer, the negative effect of aneuploidy has been a subject of controversy inasmuch as studies aimed at determining the survival–DNA content relationship have reported conflicting results. We made a meta-analysis of published studies aimed at determining the prognostic effect of aneuploidy in surgically resected non-small-cell lung cancer. 35 trials have been identified in the literature. A comprehensive collection of data has been constructed taking into account the following parameters: quality of specimen, DNA content assessment method, aneuploidy definition, histology and stage grouping, quality of surgical resection and demographic characteristics of the analysed population. Among the 4033 assessable patients, 2626 suffered from non-small-cell lung cancer with aneuploid DNA content (overall frequency of aneuploidy: 0.65; 95% CI: (0.64–0.67)). The DerSimonian and Laird method was used to estimate the size effects and the Peto and Yusuf method was used in order to generate the odds ratios (OR) of reduction in risk of death for patients affected by a nearly diploid (non-aneuploid) non-small-cell lung cancer. Survivals following surgical resection, from 1 to 5 years, were chosen as the end-points of our meta-analysis. Patients suffering from a nearly diploid tumour benefited from a significant reduction in risk of death at 1, 2, 3 and 4 years with respective OR: 0.51, 0.51, 0.45 and 0.67 ($P < 10^{-4}$ for each end-point). 5 years after resection, the reduction of death was of lesser magnitude: OR: 0.87 ($P = 0.08$). The test for overall statistical heterogeneity was conventionally significant ($P < 0.01$) for all 5 end-points, however. None of the recorded characteristics of the studies could explain this phenomenon precluding a subset analysis. Therefore, the DerSimonian and Laird method was applied inasmuch as this method allows a correction for heterogeneity. This method demonstrated an increase in survival at 1, 2, 3, 4 and 5 years for patients with diploid tumours with respective size effects of 0.11, 0.15, 0.20, 0.20 and 0.21 (value taking into account the correction for heterogeneity; $P < 10^{-4}$ for each end-point). Patients who benefit from a surgical resection for non-small-cell lung cancer with aneuploid DNA content prove to have a higher risk of death. This negative prognostic factor decreases the probability of survival by 11% at one year, a negative effect deteriorating up to 21% at 5 years following surgery. © 2001 Cancer Research Campaign http://www.bjcancer.com

**Keywords**: ploidy; non-small-cell lung cancer; prognosis

Ploidy status predicts disease-free intervals and short-term survival in numerous human malignancies (Barlogie et al 1983; Friedlander et al 1984). In lung cancer, the prognostic value of ploidy is controversial. The effect of ploidy status on patient outcome has been investigated particularly in non-small-cell lung cancers (NSCLC), a group of different histologies including squamous cell carcinoma (SQC), adenocarcinoma (ADC) and large cell carcinoma (LCC). Although authors from different laboratories have suggested that patients presenting an aneuploid tumour have a shorter survival than patients presenting a nearly diploid tumour, others did not find such a difference. Many factors could explain the differences in estimating the ploidy–survival relationship. Possible interpretation could lie in the heterogeneity of the abnormal DNA patterns gathered under the common term of aneuploidy, i.e. hyperdiploidy, hypodiploidy and multiploidy (Barlogie et al, 1980). Differences in methods used to analyse DNA content could also have been responsible for the above-mentioned controversy inasmuch as some studies were founded on flow cytometry data whereas others used static cytometry. Finally, the difference might be related to the histological and clinical characteristics of the studied patient population.

We therefore made a meta-analysis of published studies aimed at determining the prognostic effect of aneuploidy in surgically resected NSCLC.

*Received 9 January 2001*
*Revised 3 April 2001*
*Accepted 9 April 2001*

*Correspondence to:* JL Pujol

### TRIALS AND METHODS

### Eligibility criteria

The most conventional definition of ploidy status is as follows: ploidy was determined for each specimen using DNA index which represents the ratio of the cell DNA content of tumour $G_{0/1}$ cells to the diploid $G_{0/1}$ peak (2n). A coefficient of variation (CV) for this DNA index (DI) has been specifically defined for each study. Thus, a DNA index = 1 defined a near diploid specimen i.e. only one peak of $G_{0/1}$ cells in the near diploid region (2n, DNA index = 1) with few $G_2M$ tumour cells in the tetraploid region (4n). Conversely, tumours presenting a DI of less than [1 – CV] (hypodiploidy) or over [1 + CV] (hyperdiploid) were classified as aneuploid NSCLCs, as were tumours sharing multiple aneuploid peaks.

To be included in this meta-analysis, studies had to fulfil the following criteria: estimation of overall survival–DNA content relationship as aim of the study; inclusion of patients suffering from histologically proven NSCLC and operated upon in an attempt at surgical resection; no anticancer treatment (neither chemotherapy nor radiotherapy) prior to surgical resection; specification of the nature of both tested specimens and methods of DNA content assessment; standard ploidy and aneuploidy definitions; clear definition of overall survival as the time from surgery to the date of death (or date of survival update for censored patients), comprehensive survival analysis including the Kaplan–Meier method (Kaplan and Meier, 1958) estimation for incomplete observations separately analysing patient groups according to the DNA content. Alternative survival analysis using actuarial survival curves were also considered as eligible for this analysis. Finally, a 2-year minimal median follow-up was required. Studies mainly devoted to the analysis of NSCLC patients which also included a small proportion of SCLC were considered as eligible depending on separate survival analyses.

Studies with one or more of the following methodological issues were not included in the meta-analysis: histology not reported; analysis of DNA content aimed at assessing cell kinetic parameters such as identification of the percent of cells in S phase rather than ploidy status; unconventional survival report (lack of estimation of survival according to time, small subgroup analysis); unknown relationship between death and lung cancer; follow-up shorter than 2 years. In addition, studies mainly aimed at analysing survival according to treatment modalities (post-operative treatment, diagnostic only surgery) were not considered as eligible.

### Selection of trials

A computerized bibliography was extracted from MEDLINE and CANCERLIT (CancerNet™) databases using medical subject headings for the following terms: lung neoplasm, lung carcinoma, non-small-cell, ploidy or DNA content, prognosis. The search for publications in any language, was carried out from 1966 to the end of 1999 inclusive. Afterwards, the manual selection of relevant studies was based upon summary analysis. The reprint of each study was carefully analysed regarding the different eligibility criteria. In addition to the above-mentioned procedure, bibliographies of selected full papers were screened in order to disclose other relevant articles.

### Collection of data

A comprehensive collection of data has been constructed taking into account the following parameters: year of publication, aim of the study, type of sampled specimens (paraffin embedded specimen *versus* fresh tissue specimens), DNA content assessment method (flow cytometry *versus* static cytometry), aneuploidy definition, histology sub-groups and stage grouping, quality of surgical resection and demographic characteristics of the analysed population.

Survivals after surgical resection, from 1 to 5 years, were chosen as the end-points of our meta-analysis. These outcomes were assessed as follows: the parameters were directly graphically measured from magnification of publication graphs. When the data were directly reported in the text, comparisons with graphic assessment were in good agreement. An attempt to contact the first author of each selected article was made in order to obtain permission to use the data and to know whether there had been any update of the study following its publication.

### Statistics

2 methods were used in order to estimate the effects of ploidy upon survival in surgically resected NSCLC patients. The Yusuf and Peto method produces odds ratio (OR) and a 95% confidence interval together with the value of the heterogeneity test (Yusuf et al, 1985). In addition, the DerSimonian and Laird method was used in order to estimate the size effects upon the different parameters and their 95% confidence intervals were calculated (DerSimonian and Laird, 1986). For this method the value and the 95% confidence interval were corrected taking into account the heterogeneity where this latter parameter was statistically significant.

### RESULTS

A total of 35 studies fulfilled the criteria of selection (Zimmerman et al, 1987; Tirindelli-Danesi et al, 1987; Ten Velde et al, 1988; Volm et al, 1988; Yamaoka et al, 1989; Cibas et al, 1989; Dazzi et al, 1990; Isobe et al, 1990; Sahin et al, 1990; Shiota et al, 1990; Miyamato et al, 1991; Mizumoto et al, 1991; Liewald et al, 1992; Ogawa et al, 1992; Filderman et al, 1992; Morkve et al, 1993; Cheon et al, 1993; Pence et al, 1993; Rice et al, 1993; Ichinose et al, 1993; Lima et al, 1993; Shimizu et al, 1993; Yu et al, 1993; Usuda et al, 1994; Salvati et al, 1994; Tanaka et al, 1995; Pujol et al, 1996; Huang et al, 1996; Nagai et al, 1996; Jeanfaivre et al, 1997; Muguerza et al, 1997; Kolodzeijski et al, 1997; Graziano et al, 1997; Dalquen et al, 1997; Asamura et al, 1999; (Table 1)). Among the 4033 assessable patients, 2626 suffered from NSCLC with aneuploid DNA content (overall frequency of aneuploidy: 0.65; 95% Cl: (0.64–0.67)). All studies used flow cytometry measurement of DNA content except 2 which were based on static cytometry. 15 studies were prospectively conducted whereas 16 retrospectively investigated specimens from tumour banks. For the 4 remaining studies the nature of the studies population was unknown. NSCLC was the targeted population for all studies. However, three studies including less than 10% SCLC among a large NSCLC population where also taken into account (Ogawa et al, 1992; Usuda et al, 1994; Pujol et al, 1996). These SCLC cases represented 0.4% of the whole population.

Some studies reporting ploidy in NSCLC were considered as being outside the scope of this meta-analysis. 4 studies could not be selected owing to the lack of overall survival data although they appropriately reported disease-free (or relapse-free) survival (Volm et al, 1988; Carp et al, 1992; Costa et al, 1996; Roberts et al, 1998). 3 studies aimed at determining the prognostic effect of DNA content characteristics other than the classical dichotomy diploidy–aneuploidy. These characteristics targeted as prognostic factors were: type of aneuploidy patterns (Bunn et al, 1983), percent of cells in the aneuploid $G_1$ peak (Van Bodegom et al, 1989) and hypodiploid/hypertetraploidy patterns (Stipa et al, 1993). One study (Del Carlo Bernardi et al, 1997) defined diploid tumours as tumours presenting a DI $\leq$ 1.2 a feature shared by hypodiploid tumours (Barlogie et al, 1980; Stipa et al, 1993). 3 publications were preliminary reports of DNA content–survival relationship (Salvati et al, 1989; Ichinose et al, 1991; Ogawa et al, 1991) but otherwise subsequently reported by the respective groups (Ogawa et al, 1992; Ichinose et al, 1993; Salvati et al,

*© 2001 Cancer Research Campaign* 

**Table 1** Characteristics of ploidy studies in non-small-cell lung cancer

| Publications | Type of samples[1] | Method of analysis[2] | Study design | No. of evaluable patients | Mean age | % Aneuploidy | % ADC | % SQC | %LCC |
|---|---|---|---|---|---|---|---|---|---|
| Zimmerman et al, 1987 | PES | FCM | Retrospective | 100 | UNK | 0.45 | 0.40 | 0.48 | 0.12 |
| Tirindelli et al, 1987 | FTS | FCM | Prospective | 63 | UNK | 0.51 | UNK | UNK | UNK |
| Ten velde et al, 1988 | PES | FCM | Retrospective | 67 | UNK | 0.66 | 0.09 | 0.88 | 0.03 |
| Volm et al, 1988 | FTS | FCM | Prospective | 175 | UNK | 0.85 | 0.28 | 0.57 | 0.15 |
| Yamaoka et al, 1989 | PES | FCM | UNK | 179 | UNK | 0.77 | UNK | UNK | UNK |
| Cibas et al, 1989 | PES | FCM | Prospective | 93 | 60 | 0.85 | 1 | 0 | 0 |
| Dazzi et al, 1990 | PES | FCM | Retrospective | 136 | UNK | 0.70 | 0.22 | 0.64 | 0.14 |
| Isobe et al, 1990 | PES | FCM | Prospective | 80 | UNK | 0.75 | 0.53 | 0.48 | 0 |
| Sahin et al, 1990 | PES | FCM | Prospective | 146 | 59 | 0.58 | 0.51 | 0.38 | 0.11 |
| Shiota et al, 1990 | PES | FCM | UNK | 94 | UNK | 0.57 | UNK | UNK | UNK |
| Miyamoto et al, 1991 | PES | FCM | Retrospective | 83 | UNK | 0.77 | 0.52 | 0.48 | 0 |
| Mizumoto et al, 1991[2] | PES/FTS | FCM | UNK | 155 | UNK | 0.68 | 1 | 0 | 0 |
| Liewald et al, 1992 | PES/FTS | FCM | Prospective | 99 | 61 | 0.48 | 0.41 | 0.48 | 0.10 |
| Ogawa et al[3], 1992 | PES | FCM | Retrospective | 56 | 62 | 0.63 | 0.66 | 0.27 | 0.05 |
| Filderman et al, 1992 | PES | FCM | Retrospective | 44 | 62 | 0.20 | 0.61 | 0.34 | 0.05 |
| Morkve et al, 1993 | PES | FCM | Retrospective | 112 | 62.6 | 0.45 | 0.49 | 0.45 | 0.06 |
| Cheon et al, 1993 | PES | FCM | Retrospective | 56 | 58 | 0.46 | 0.29 | 0.64 | 0.07 |
| Pence et al, 1993 | FTS | SCM | Prospective | 61 | 63 | 0.64 | 0.46 | 0.43 | 0.11 |
| Rice et al, 1993 | FTS | FCM | Prospective | 272 | 65.5 | 0.82 | 0.42 | 0.37 | 0.21 |
| Ichinose et al, 1993 | PES | FCM | Prospective | 151 | 64.3 | 0.65 | 0.62 | 0.36 | 0.01 |
| Yu et al, 1993 | PES | FCM | Retrospective | 47 | 61 | 0.66 | 0.13 | 0.49 | 0.38 |
| Lima et al, 1993 | PES | FCM | Retrospective | 45 | 61.2 | 0.60 | 1 | 0 | 0 |
| Shimizu et al, 1993 | UNK | UNK | Retrospective | 82 | UNK | 0.80 | UNK | UNK | UNK |
| Usuda et al[3], 1994 | PES | FCM | UNK | 65 | UNK | 0.72 | 0.60 | 0.32 | 0.03 |
| Salvati et al, 1994 | FTS | FCM | Prospective | 142 | UNK | 0.58 | UNK | UNK | UNK |
| Tanaka et al, 1995 | PES | FCM | Retrospective | 160 | UNK | 0.68 | 1 | 0 | 0 |
| Pujol et al[3], 1996 | FTS | SCM | Prospective | 137 | 62 | 0.58 | 0.25 | 0.62 | 0.03 |
| Huang et al, 1996 | PES | FCM | Retrospective | 87 | UNK | 0.72 | 1 | 0 | 0 |
| Nagai et al, 1996 | PES | FCM | Prospective | 340 | UNK | 0.65 | 0.57 | 0.38 | 0.05 |
| Jeanfaivre et al, 1997 | FTS | FCM | Retrospective | 61 | 60 | 0.82 | 0 | 1 | 0 |
| Muguerza et al, 1997 | PES/FTS | FCM | Prospective | 124 | 62 | 0.60 | 0.28 | 0.63 | 0.09 |
| Kolodziejski et al, 1997 | PES | FCM | Retrospective | 207 | 55.1 | 0.42 | 0 | 1 | 0 |
| Graziano et al, 1997 | PES | FCM | Retrospective | 145 | UNK | 0.70 | UNK | UNK | UNK |
| Dalquen et al, 1997 | PES | FCM | Prospective | 97 | UNK | 0.76 | UNK | UNK | UNK |
| Asamura et al, 1999 | PES | FCM | Prospective | 72 | 61.7 | 0.51 | 1 | 0 | 0 |

[1]PES: paraffin embedded specimen; FTS: fresh tissue specimens; [2] FCM: flow cytometry; SCM: static cytometry; ADC: adenocarcinoma, SQC: squamous cell carcinoma; LCC: large cell carcinoma; UNK: unknown.
[3]Three studies included less than 10% small cell lung cancers.

© 2001 Cancer Research Campaign

1994). 7 publications aimed at determining the relationship between ploidy status and other clinical and biological features such as *c-erb*B-2 and L-*myc* expressions (Chiba et al, 1993), Ki-67 growth-fraction evaluation (Simony et al, 1990), stage grouping (Ikeda et al, 1995), S-phase (Granone et al, 1993; Visakorpi et al, 1995), p53 gene mutations (Casson et al, 1994), or bromodeoxyuridine labelling (Shimosato et al, 1989). In these studies the survival data were neither exhaustively reported nor considered as the primary end-point of the experiment. Finally one study aimed at describing a new static cytometric method and secondarily reported descriptive relapse data (Blondal and Bengtson, 1981).

### Peto and Yusuf

Patients suffering from nearly diploid tumours benefited from a significant reduction in risk of death at 1, 2, 3 and 4 years with respective OR: 0.51, 0.51, 0.45 and 0.67 ($P < 10^{-4}$ for each end-point; Figures 1–4). 5 years after resection, the reduction of death was of lesser magnitude: OR: 0.87 ($P = 0.08$; Figures 5). The test for overall statistical heterogeneity was conventionally significant ($P < 0.01$; Table 2) for all 5 end-points, however. None of the recorded characteristics of the studies were able to explain this phenomenon precluding a subset analysis.

### DerSimonian and Laird

The DerSimonian and Laird method was applied inasmuch as this method allows a correction for heterogeneity. This method demonstrated an increase in survival at 1, 2, 3, 4 and 5 years for patients with diploid tumours with respective size effects of 0.11, 0.15, 0.20, 0.20 and 0.21 (value taking into account the correction for heterogeneity; $P < 10^{-4}$ for each end-point).

### DISCUSSION

DNA content analysis has been proposed to assess cell kinetics insofar as the DNA histogram allows the identification of the percent of cells in S phase. However, the assessment of S phase is frequently hampered by the overlap between aneuploid tumour-cell population and diploid non-malignant cell population. Thus, in human malignancies, DNA content analysis is mainly used to evaluate the occurrence of aneuploid cell population, an abnormality known to characterize malignant cells (Barlogie et al, 1980). The study of cell kinetics has been proposed as a reliable prognostic parameter in human malignancies (Barlogie et al, 1980; Latreille et al, 1980; Friedlander et al, 1984). Aneuploidy can predict short-term survival in many solid tumours (Wolley et al, 1982; Auer et al, 1984; Friedlander et al, 1984; Bondeson et al, 1986; Matsura et al, 1986). In lung cancer the prognostic significance of an abnormal DNA content might be regarded, in a logical way, as consistent with knowledge of tumour biology for this disease. However, published studies have generated conflicting results and currently the negative effect of aneuploidy in NSCLC is still controversial.

In this study we used a meta-analytic approach to this question in an attempt to determine the ploidy-survival relationship and, depending on its existence, the magnitude of the effect. One may hypothesize that, due to the nature of the herein meta-analysis based on published data, a possible bias was introduced as our procedure did not allow the disclosure of unpublished studies. A direct comparison of meta-analysis on medical literature and meta-analysis on individual patient data has been made in the setting of chemotherapy in ovarian cancer (Stewart and Parmar, 1993). This study suggested possible differences in estimated treatment effect due to patient exclusions and shorter length of follow-up in the former technique. However, the meta-analysis reported here takes into account 35 studies which as a whole included over 4000 patients suffering from NSCLC. A long-term survival end-point has been chosen to avoid overestimation of effect by a short follow-up period. No apparent effect of epoch appeared when year of publication was taken into account. The proportion of tumours presenting an aneuploid DNA content is set at 64% and was reported homogeneously throughout the panel of studies analysed. One can therefore speculate that publication bias could be minimal although remaining unknown.

The different study designs are similar. However, statistical heterogeneity has been detected by both meta-analysis methods regarding survival estimation. Using the DerSimonian and Laird

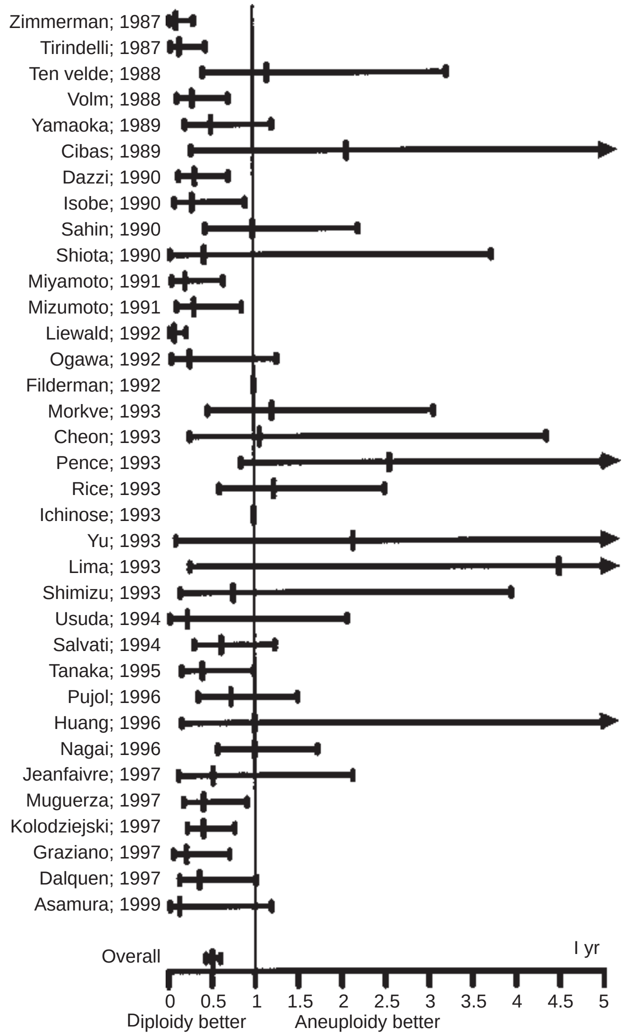

**Figure 1** Odds ratio and 95% confidence interval of mortality at 1 year for patients operated upon for a NSCLC with nearly diploid DNA content ($P < 10^{-4}$). Results are expressed as individual and overall Ors; vertical bar, and their respective 95% confidence intervals; horizontal bar. ORs lower than 1 indicate a reduction in risk of death for patients affected by a diploid NSCLC. See in Table 2, test for heterogeneity

© 2001 Cancer Research Campaign

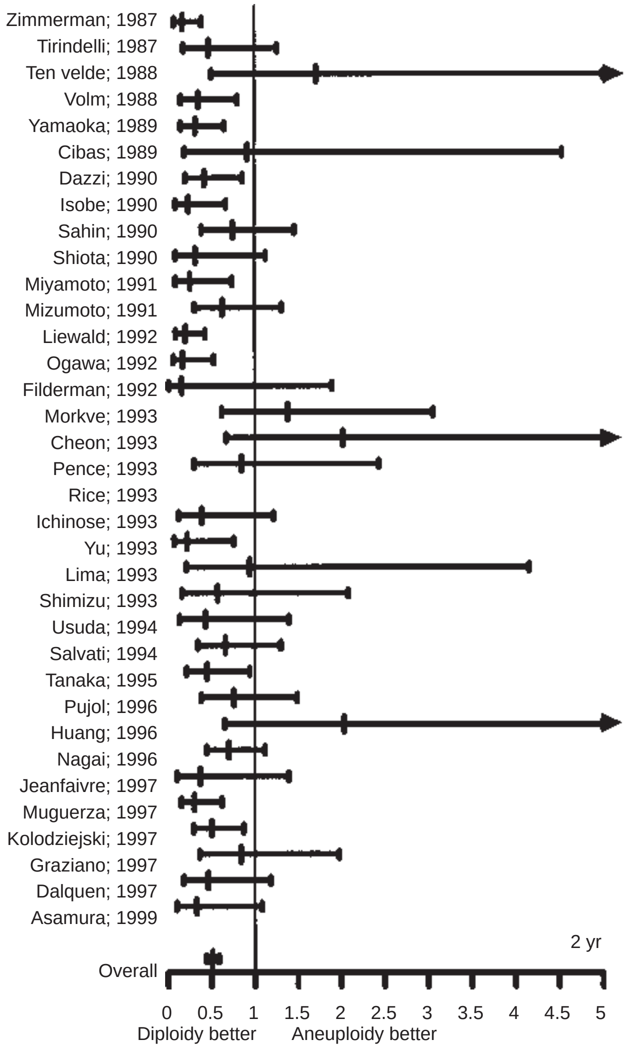

**Figure 2** Odds ratio and 95% confidence interval of mortality at 2 years for patients operated upon for a NSCLC with nearly diploid DNA content (symbols as in Figure 1; *P* = < $10^{-4}$)

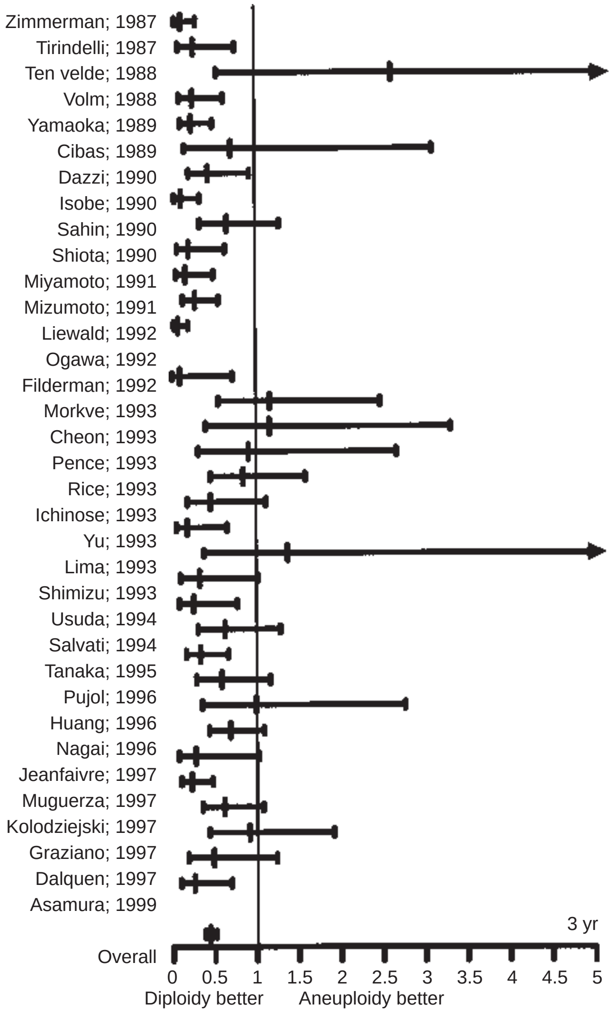

**Figure 3** Odds ratio and 95% confidence interval of mortality at 3 years for patients operated upon for a NSCLC with nearly diploid DNA content (symbols as in Figure 1; *P* = < $10^{-4}$)

method this heterogeneity has been taken into account. In addition, the magnitude of the survival effect has been set by the latter method as a complementary estimation of prognosis aside from the classical OR determination using the Peto and Yusuf method. There is, so far, no definitive explanation for the heterogeneity. Among the different hypotheses, we investigated whether the method of analysis could be responsible for the difference. Flow cytometry is considered as the standard method to analyse DNA content histograms after propidium iodine staining of a single-cell suspension. This method takes into account thousands of cells. Static cytometry has been proposed as an alternative method. It analyses cytological prints of the tumour after Feulgen staining. This second method only analyses a few hundred cells. Although this number is lower than the one analysed by flow cytometry, the computer-assisted image processor is able to distinguish between tumour cells to be analysed and tumour-infiltrating lymphocytes. In addition, studies carried out in order to compare the 2 methods demonstrated the reliability of ploidy analysis using static cytometry (Friedlander et al, 1984; Oud et al, 1989). Thus, static computer-assisted cytometry is considered as a reliable means of analysing ploidy in situ. In this meta-analysis, both studies based on this technique produced results in accordance with the final survival–aneuploidy relationship as generated by the meta-analysis. Neither the nature of the samples, nor the histology or stage of the studied population could be a putative reason for the heterogeneity. In particular, published studies seldom reported on difference in aneuploidy based on histological subgroup (i.e. adenocarcinoma, SQC or LCC). Consequently, it is not possible to determine whether or not difference in NSCLC histology from one study to the other could explain the statistical heterogeneity. In addition, the distribution of aneuploid and diploid tumours by stage has been recorded in our database whenever the data was reported by each study. Out of the 18 studies with detail analysis by stages, the respective percentage of aneuploid tumours in stage I, stage II and stage III was 58, 57 and 70%. Therefore, it seems unlikely that the statistical heterogeneity regarding survival estimation of the whole meta-analysis, belongs to the distribution of stage grouping of the different accrued populations, insofar as the frequency of aneuploidy did not differ from one stage group to the other.

The type of sample analysed could hardly be considered as a possible inductor of heterogeneity across the panel of studies

*© 2001 Cancer Research Campaign*

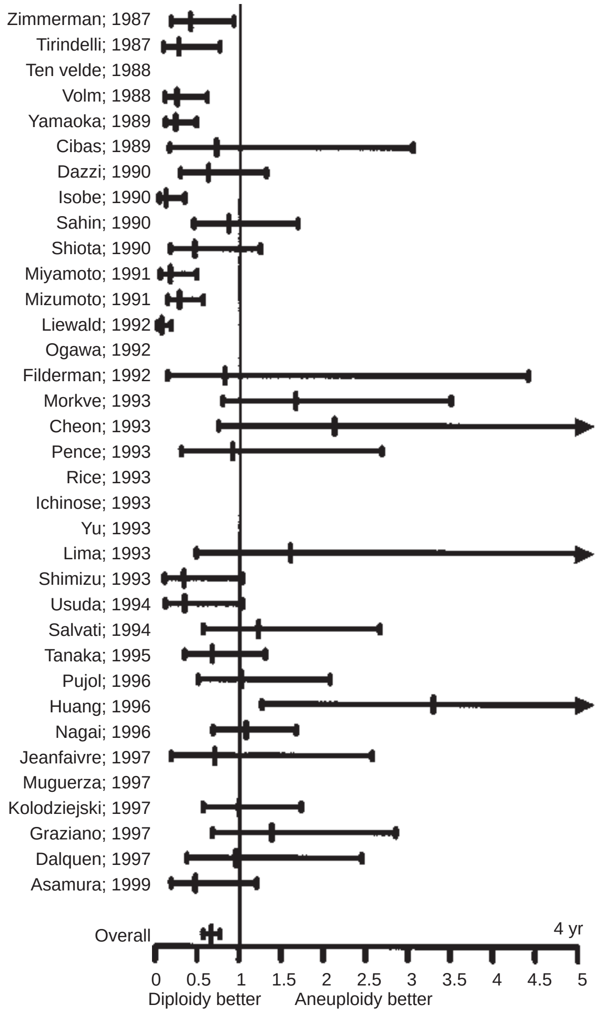

**Figure 4** Odds ratio and 95% confidence interval of mortality at 4 years for patients operated upon for a NSCLC with nearly diploid DNA content (symbols as in Figure 1; $P = < 10^{-4}$)

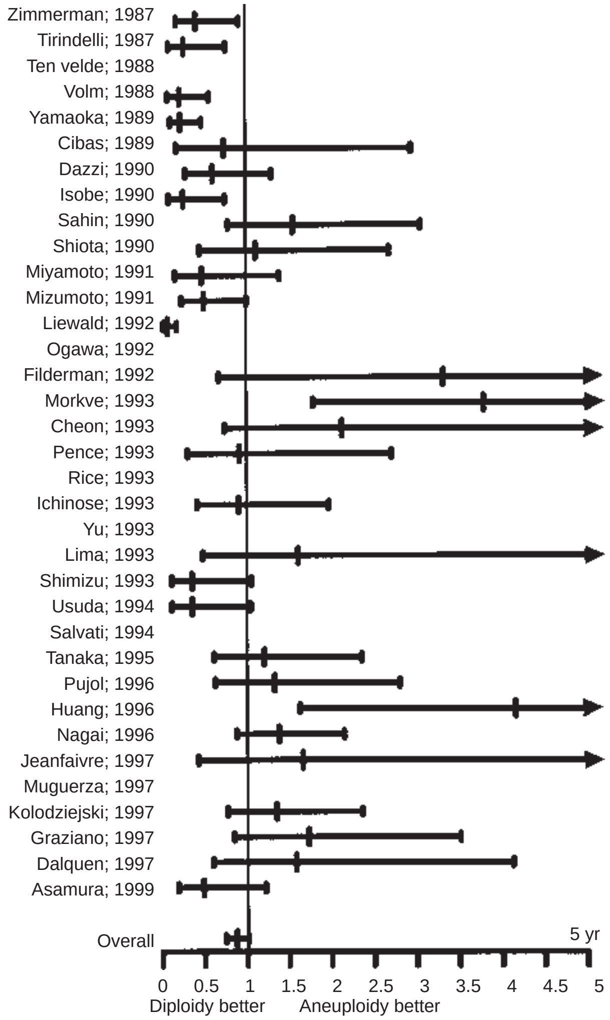

**Figure 5** Odds ratio and 95% confidence interval of mortality at 5 years for patients operated upon for a NSCLC with nearly diploid DNA content ($P < 10^{-4}$) (symbols as in Figure 1; $P = 0.08$).

**Table 2** Results of Peto and Yusuf meta-analysis

| Endpoint | OR | Lower | Upper | *P* | –logOR/ σ(logOR) | Heterogeneity | Degree of freedom | *P* heterogeneity |
|---|---|---|---|---|---|---|---|---|
| 1 y | 0.5130 | 0.4323 | 0.6087 | < 0.0001 | 6.74 | Q = 76.60 | 32 | *P* < 0.01 |
| 2 y | 0.5092 | 0.4387 | 0.5837 | < 0.0001 | 9.24 | Q = 59.00 | 33 | *P* < 0.01 |
| 3 y | 0.4461 | 0.3872 | 0.5139 | < 0.0001 | 11.17 | Q = 87.50 | 33 | *P* < 0.01 |
| 4 y | 0.6666 | 0.5731 | 0.7753 | < 0.0001 | 5.26 | Q = 119.2 | 29 | *P* < 0.01 |
| 5 y | 0.8742 | 0.7510 | 1.0170 | 0.08 | 1.74 | Q = 123.9 | 28 | *P* < 0.01 |

Odds ratios indicate the reduction of risk of death for patients presenting a nearly diploid tumour.

**Table 3** Results of Dersimonian and Laird meta-analysis

| Endpoint | Diploidy | Aneuploidy | Y* | Y : 95% confidence interval | *P* | Heterogeneity | Degrees of freedom | *P* heterogeneity |
|---|---|---|---|---|---|---|---|---|
| 1 y | *P* = 0.871 | *P* = 0.763 | 0.11 | 0.07; 0.15 | < 0.0001 | Q = 111.3 | 32 | < 0.005 |
| 2 y | *P* = 0.739 | *P* = 0.590 | 0.15 | 0.10; 0.20 | < 0.0001 | Q = 78.42 | 33 | < 0.005 |
| 3 y | *P* = 0.660 | *P* = 0.460 | 0.20 | 0.14; 0.26 | < 0.0001 | Q = 117.62 | 33 | < 0.005 |
| 4 y | *P* = 0.603 | *P* = 0.400 | 0.20 | 0.13; 0.27 | < 0.0001 | Q = 142.58 | 29 | < 0.005 |
| 5 y | *P* = 0.604 | *P* = 0.391 | 0.21 | 0.15; 0.28 | < 0.0001 | Q = 105.53 | 28 | < 0.005 |

*Size effect according to DerSimonian and Laird's method. Corrected values taking into account a significant heterogeneity.

*© 2001 Cancer Research Campaign*

owing to the fact that there is a good agreement between the DNA histograms obtained from fresh tissue and paraffin-embedded specimens where flow cytometry has been done in parallel (Hedley et al, 1983). A possible hidden explanation of the statistical heterogeneity lies in the well-established diversity of DNA content histograms and growth fraction estimations in a given specimen (Sara et al, 1986; Oud et al, 1989; Simony et al, 1990; Stipa et al, 1993). Besides this heterogeneity, one can emphasize that aneuploidy is a common terminology designating different histogram patterns. Multiploidy and hypodiploidy might be underestimated in some specimens. In multiple myeloma and breast cancer attention has been paid to the occurrence of these 2 particular ploidy status (Coulson et al, 1984; Smith et al, 1986). A similar observation has been made in lung cancer in which hypodiploidy seems to indicate a particularly poor outcome (Pujol et al, 1996).

Hitherto, the prognosis of patients with surgically resected NSCLC is mainly determined using two main prognostic variables, i.e. the stage of the disease and the performance status. During the past decade, the search for genetic markers of NSCLC has emerged in an attempt to predict poor outcome of this disease and to help the clinician in deciding whether there is a case for adjuvant chemotherapy. Abnormal nuclear content has long been known as a conclusive marker of malignancy and was found in increased frequency in solid tumours. It is noteworthy that the 65% overall frequency of aneuploidy, as observed in this meta-analysis is consistent with the 67% frequency reported in 4941 patients suffering from various human malignancies (Barlogie et al, 1983). During the 1990s, after an initial period of enthusiasm for aneuploidy as a putative prognostic marker in NSCLC, the interest seemed to decrease. This might be explained partially by the fact that conflicting results arose from different studies. However, the meta-analysis described above seems to provide additional clues in favour of a prognostic effect of aneuploidy in this disease. DNA content analysis provides a unique insight into the cellular heterogeneity and the genotypic instability of NSCLC. Chromosomal instability leads to abnormal regulation of gene expression and aneuploidy. This latter characteristic might be a critical factor for phenotypic diversification towards a metastatic phenotype (Nicolson, 1987).

Notwithstanding the heterogeneity across the studies, the herein meta-analysis allows the conclusion that patients who benefit from a surgical resection for NSCLC with aneuploid DNA content prove to have a higher risk of death. The survival probability for patients having an aneuploid tumour is decreased by 11% at one year, a negative effect deteriorating up to 21% at 5 years following surgery.

### ACKNOWLEDGEMENTS

The authors wish to thank Mrs Jo Baïssus for help in preparing the manuscript. Supported by a grant from the French League Against Cancer (Hérault committee).

### REFERENCES

Asamura H, Ando M, Matsuno Y and Shimosato Y (1999) Histopathologic prognostic factors in resected adenocarcinomas. *Chest* **115**: 1018–1024

Auer G, Eriksson E, Azavedo E, Caspersson T and Wallgren A (1984) Prognostic significance of nuclear DNA content in mammary adenocarcinomas in humans. *Cancer Res* **44**: 394–396

Barlogie B, Drewinko B, Schumann J, Gohde W, Dosik G, Latreille J, Johnston DA and Freireich EJ (1980) Cellular DNA content as a marker of neoplasia in man. *Am J Med* **69**: 195–203

Barlogie B, Raber MN, Shumann J, Johnson TS, Drewinki B, Swartzendruber DE, Göhde W, Andreeff M and Feireich EJ (1983) Flow cytometry in clinical cancer research. *Cancer Res* **43**: 3982–3997

Blondal T and Bengtsson A (1981) Nuclear DNA measurements in squamous cell carcinoma of the lung: a guide for prognostic evaluation. *Anticancer Res* **1**: 79–86

Bondeson L, Azavedo E, Bondeson AG, Caspersson T and Ljungberg O (1986) Nuclear DNA content and behavior of oxyphil thyroid tumors. *Cancer* **58**: 672–675

Bunn PA, Carney DN, Gazdar AF, Whang-Peng J and Matthews MJ (1983) Diagnostic and biological implications of flow cytometry DNA content analysis in lung cancer. *Cancer Res* **43**: 5026–5032

Carp NZ, Ellison DD, Brophy PF, Watts P, Chang MC and Keller SM (1992) DNA content in correlation with post-surgical stage in non-small-cell lung cancer. *Ann Thorac Surg* **53**: 680–683

Casson AG, McCuaig S, Craig I, Ayed A, Inculet R, Kerkvliet N and O'Malley F (1994) Prognostic value and clinicopathologic correlation of p53 gene mutations and nuclear DNA content in human lung cancer: a prospective study. *J Surg Oncol* **56**: 13–20

Cheon SH, Sohn HY, Chang J, Kim SK, Ko EH, Kim SK, Lee WY, Lee DY, Shin DH, Jeong ET and Chung HT (1993) Flow cytometric analysis of DNA ploidy in primary non-small-cell carcinoma of the lung in Korea. *Yonsei Med J* **34**: 365–370

Chiba W, Sawai S, Hanawa T, Ishida H, Matsui T, Kosaba S, Watanabe S, Hatakenaka R, Matsubara Y, Funatsu T, Ikeda S, Kinoshita M and Matsumoto H (1993) Correlation between DNA content and amplification of oncogenes (c-myc, L-myc, c-erbB-2) and correlation with prognosis in 143 cases of resected lung cancer. *Jpn J Cancer Chemother* **20**: 824–827

Cibas ES, Melamed MR, Zaman, MB and Kimmel M (1989) The effect of tumor size and tumor cell DNA content on the survival of patients with stage I adenocarcinoma of the lung. *Cancer* **63**: 1552–1556

Costa A, Silverstrini R, Mochen C, Lequaglie C, Boracchi P, Faranda A, Vessecchia G and Ravasi G (1996) p53 expression, DNA ploidy and S-pase cell fraction in operable locally advanced non-small-cell lung cancer. *Br J Cancer* **73**: 914–919

Coulson BP, Thornthwaite JT, Weelley TW, Sugarbaker EV and Seckinger D (1984) Prognostic indicators including DNA histogram type, receptor content, and staging related to human breast cancer patient survival. *Cancer Res* **44**: 4187–4196

Dalquen P, Moch H, Feichter G, Lehmann M, Solèr M, Stulz P, Jordan P, Torhorst J, Mihatsch MJ and Sauter G (1997) DNA aneuploidy, S-phase fraction, nuclear p53 positivity, and survival in non-small-cell lung carcinoma. *Virchows Arch* **431**: 173–179

Dazzi H, Thatcher N, Hasleton PS and Swindell R (1990) DNA analysis by flow cytometry in non-small-cell lung cancer: relationship to epidermal growth factor receptor, histology, tumour stage and survival. *Respir Med* **84**: 217–223

Del Carlo Bernardi F, Antonangelo L, Beyruti R, Takagaki T, Nascimento Saldiva PH and Capelozzi VL (1997) A prognostic model of survival in surgically resected squamous cell carcinoma of the lung using clinical, pathologic and biologic markers. *Mod Pathol* **10**: 992–1000

DerSimonian R and Laird N (1986) Meta-analysis in clinical trials. *Control Clin Trials* **7**: 177–188

Filderman AE, Silvestri GA, Gatsonis C, Luthringer DJ, Honig J and Flynn SD (1992) Prognostic significance of tumor proliferative fraction and DNA content in stage I non-small-cell lung cancer. *Am Rev Respir Dis* **146**: 707–710

Friedlander ML, Hedley DW and Taylor IW (1984a) Clinical and biological significance of aneuploidy in human tumours. *J clin Pathol* **37**: 961–974

Friedlander ML, Hedley DW, Taylor IW, Russell P, Coastes AS and Tattersall MHN (1984b) Influence of cellular DNA content on survival in advanced ovarian cancer. *Cancer Res* **44**: 397–400

Granone P, Cardillo G, Rumi E, D'Ugo D, Rumi C, Ciletti S, Margaritora S, Terribile D and Picciocchi A (1993) DNA flow cytometric analysis in patients with operable non-small-cell lung carcinoma. *Eur J Cardio-thorac Surg* **7**: 351–355

Graziano SL, Tatum AH, Gonchoroff NJ, Newman NB and Kohman LJ (1997) Blood group antigen A and flow cytometric analysis in resected early-stage non-small-cell lung cancer. *Clin Cancer Res* **3**: 87–93

Hedley DW, Friedlander ML, Taylor IW, Rugg CA and Musgrove EA (1983) Method for analysis of cellular DNA content of paraffin-embedded pathological material using flow cytometry. *J Histochem Cytochem* **31**: 1333–1335

© 2001 Cancer Research Campaign

Huang MS, Colby TV, Therneau TM, Daly RC and Gonchoroff NJ (1996) DNA ploidy and protein content in bronchioloalveolar carcinoma multi-variable flow cytometry. *Cytometry* **26**: 253–259

Ichinose Y, Hara N, Ohta M, Motohiro A, Kuda T and Aso H (1991) Postoperative adjuvant chemotherapy in non-small-cell lung cancer: prognostic value of DNA ploidy and post-recurrent survival. *J Surg Oncol* **46**: 15–20

Ichinose Y, Hara N, Ohta M, Yano T, Maeda K, Asoh H and Katsuda Y (1993) Is T factor of the TNM staging system a predominant prognostic factor in pathologic stage I non-small-cell lung cancer? *J Thorac Cardiovasc Surg* **106**: 90–94

Ikeda N, McAulay C, Lam S, Garner DM, Payne PW, Kato H, Konaka C and Palcic B (1995) Use of high-resolution cytometry in predicting the biologic behavior of T1 adenocarcinoma of the lung. *Analyt Quant Cytol Histol* **17**: 69–74

Isobe H, Miyamoto H, Shimizu T, Haneda H, Hashimoto M, Inoue K, Mizuno S and Kawakami Y (1990) Prognostic and therapeutic significance of the flow cytometric nuclear DNA content in non-small-cell lung cancer. *Cancer* **65**: 1391–1395

Jeanfaivre T, Chassevent A, Geslin J, Larra F and Tuchais E (1997) Valeur pronostique de la cytometrie en flux dans les cancers bronchiques epidermoides. Etude Retrospective a propos de 61 cas. *Bull Cancer* **84**: 597–602

Kaplan EL and Meier P (1958) Nonparametric estimation from incomplete observations. *J Am Stat Assoc* **53**: 457–481

Kolodziejski L, Niezabitowski A and Gasinska A (1997) Clinical and flow cytometric prognostic factors in surgically treated squamous cell lung cancer. *Lung Cancer* **16**: 173–182

Latreille J, Barlogie B, Dosik G, Johnston DA, Drewinko B and Alexanian R (1980) Cellular DNA content as a marker of human multiple myeloma. *Blood* **55**: 403–408

Liewald F, Hatz R, Storck M, Orend KH, Weiss M, Wulf G, Valet G and Sunder-Plassmann L (1992) prognostic value of deoxyribonucleic acid aneuploidy in primary non-small-cell lung carcinomas and their metastases. *J Thorac Cardiovasc Surg* **104**: 1476–1482

Lima C, Matsui K, Kitagawa M, Mizushima Y, Noto H and Miwa A (1993) Analysis of prognostic factors in patients with resected peripheral T1 adenocarcinoma of the lung. *Path Res Pract* **189**: 1149–1153

Matsura H, Sugimachi K, Ueo H, Kuwano H, Koga Y and Okamura T (1986) Malignant potentiality of squamous cell carcinoma of the esophagus predictable by DNA content. *Cancer* **57**: 1810–1814

Miyamoto H, Harada M, Isobe H, Akita HD, Haneda H, Yamaguchi E, Kuzumaki N and Kawakami Y (1991) Prognostic value of nuclear DNA content and expression of the ras oncogene product in lung cancer. *Cancer Res* **51**: 6346–6350

Mizumoto T, Tokui T, Kusagawa H, Sato T, Wada K, Den T, Kimura M, Namikawa S and Kusagawa M (1991) DNA analysis of resected pulmonary adenocarcinoma. *Nippon Kyobu Geka Gakkai Zasshi* **39**: 1870–1875

Morkve O, Halvorsen OJ, Skjaerven R, Stangeland L, Gulsvik A and Laerum OD (1993) Prognostic significance of p53 protein expression and DNA ploidy in surgically treated non-small-cell lung carcinomas. *Anticancer Res* **13**: 571–578

Muguerza JM, Diez M, Torres AJ, Lopez-Asenjo JA, Picardo AL, Gomez A, Hernando F, Cayon R, Granell J and Balibrea JL (1997) Prognostic value of flow cytometric DNA analysis in non-small-cell lung cancer: rationale of sequential processing of frozen and paraffin-embedded tissue. *World J Surg* **21**: 323–329

Nagai S, Chiba W, Ikeda S, Matsumoto H, Fujimoto T, Ishida H, Wazawa H, Hanawa T, Yamashita N, Yasuda Y, Matsubara Y, Hatakenaka R and Funatsu T (1996) Flow cytometric analysis of the DNA content of resected non-small-cell lung cancer with reference to long–term follow-up. *Jpn J Cancer Chemother* **23**: 130–134

Nicolson, G.L (1987) Tumor cell instability, diversification, and progression to the metastatic phenotype: from oncogene to oncofetal expression. *Cancer Res* **47**: 1473–1487

Ogawa JI, Iwazaki M, Tsurumi T, Inoue H and Shohtsu A (1991) Prognostic implications of DNA histogram, DNA content, and histologic changes of regional lymph nodes in patients with lung cancer. *Cancer* **67**: 1370–1376

Ogawa JI, Tsurumi T, Inoue H and Shohtsu A (1992) Relationship between tumor DNA ploidy and regional lymph node changes in lung cancer. *Cancer* **69**: 1688–1695

Oud PS, Pahlplatz MMM, Beck JLM, Wiersma–Van Tilburg A, Wagenaar SJ and Vooijs GP (1989) Image and flow cytometry of small cell carcinoma of the lung. *Cancer* **64**: 1304–1309

Pence JC, Kerns BJM, Dodge RK and Iglehart JD (1993) Prognostic significance of the proliferation index in surgically resected non-small-cell lung cancer. *Arch Surg* **128**: 1382–1390

Pujol JL, Simony J, Jolimoy G, Jaffuel D, Demoly P, Quantin X, Marty-Ane C, Boher JM, Charpentier R and Michel FB (1996) Hypodiploidy, Ki-67 growth fraction and prognosis of surgically resected lung cancers. *Br J Cancer* **74**: 964–970

Rice TW, Bauer TW, Gephardt GN, Medendorp SV, McLain DA and Kirby TJ (1993) Prognostic significance of flow cytometry in non-small-cell lung cancer. *J Thorac Cardiovasc Surg* **106**: 210–217

Roberts HL, Komaki R, Allen P and EI-Naggar AK (1998) Prognostic significance of DNA content in stage I adenocarcinoma of the lung. *Int J Radiation Oncology Biol Phys* **41**: 573–578

Sahin AA, Ro JY, EI-Naggar AK, Lee JS, Ayala AG, Teague K and Hong WK (1990) Flow cytometric analysis of the DNA content of non-small-cell lung cancer. *Cancer* **65**: 530–537

Salvati F, Teodori L, Gagliardi L, Signora M, Aquilini M and Storniello G (1989) DNA flow cytometric studies of 66 human lung tumors analyzed before treatment. Prognostic implications. *Chest* **96**: 1092–1098

Salvati F, Teodori L, Trinca ML, Pasquali-Lasagni R and Gohde W (1994) The relevance of flow-cytometric DNA content in the evaluation of lung cancer. *J Cancer Res Clin Oncol*, **120**: 233–239

Sara A and EI-Naggar AK (1991) Intratumoral DNA content variability: a study of non-small-cell lung cancer. *Am J Clin Pathol* **96**: 311–317

Shimizu J, Watanabe Y, Oda M, Hayashi Y, Ota Y, Morita K and Arano Y (1993) Results of surgical treatment of stage I lung cancer. *Nippon Geka gakkai Zasshi* **94**: 505–510

Shimosato Y, Asamura H, Yoshida K, Noguchi M, Nakajima T and Mukai K (1989) Factors possibly affecting the degree of malignancy in adenocarcinoma of the lung. DNA content and Bromodeoxyuridine (Brdu) labeling index. *Chest* **S96**: 37–38

Shiota T, Konishi T, Kosaba S, Mitsuoka A, Matsubara Y, Hatakenaka R, Funatsu T, Ikeda S, Matsumoto H and Ishigami F (1990) Flow cytometric analysis of cellular DNA content of lung cancer with reference to survival. *Nippon Kyobu Geka Gakkai Zasshi* **38**: 2364–2369

Simony J, Pujol JL, Radal M, Ursule E, Michel FB and Pujol H (1990) In situ evaluation of growth fraction determined by monoclonal antibody Ki-67 and ploidy in surgically resected non-small-cell lung cancers. *Cancer Res* **50**: 4382–4387

Smith L, Barlogie B and Alexanian R (1986) Biclonal and hypodiploid multiple myeloma. *Am J Med* **80**: 841–843

Stewart LA and Parmar MK (1993) Meta-analysis of the literature or of individual patient data: is there a difference? *Lancet* **341**: 418–422

Stipa S, Tirenidelli-Danesi D, Modini C, Cicconetti F, Mauro F, Schillici A, Mecozzi A, Nicolanti V, Stipa F, Mancini M, Bangrazi C, and Botti C (1993) The importance of heterogeneity and of multiple site sampling in the prospective determination of deoxyribonucleic acid flow cytometry. *Surg Gynecol Obstet* **176**: 427–434

Tanaka I, Masuda R, Furuhata Y, Inoue M, Fujiwara M and Takemura T (1995) Flow cytometric analysis of the DNA content of adenocarcinoma of the lung, especially for patients with stage I disease with long term follow-up. *Cancer* **75**: 2461–2465

Ten Velde GPM, Schutte B, Vermeulen A, Volovics A, Reynders MMJ and Blijham GH (1988) Flow cytometric analysis of DNA ploidy level in paraffin-embedded tissue of non-small-cell lung cancer. *Eur J Cancer Clin Oncol* **24**: 455–460

Tirindelli-Danesi D, Teodori L, Mauro F, Modini C, Botti C, Cicconetti F and Stipa S (1987) Prognostic significance of flow cytometry in lung cancer. A 5-year study. *Cancer* **60**: 844–851

Usuda K, Saito Y, Aikawa H, Sakurada A, Chen Y, Takahashi S, Kanma K, Sato M, Sagawa M and Fujimura S (1994) Flow cytometric analysis of nuclear DNA content of lung cancer. Correlation between nuclear DNA content and tumor doubling time. *J Jpn Assn Thorac Surg* **42**: 486–491

Van Bodegom PC, Baak JPA, Stroet-Van Galen C, Schipper NW, Wisse-Brekelmans ECM, Vanderschueren RGJRA and Wagenaar SS (1989) The percentage of aneuploidy cells is significantly correlated with survival in accurately staged patients with stage 1 resected squamous cell lung cancer and long term follow-up. *Cancer* **63**: 143–147

Visakorpi T, Holli K and Hakama M (1995) High cell proliferation activity determined by DNA flow cytometry and prognosis in epidermoid lung carcinoma. *Acta Oncol* **34**: 605–609

Volm M, Bak M, Hanh EW, Mattern J and Weber E (1988a) DNA and S-phase distribution and incidence of metastasis in human primary lung carcinoma. *Cytometry* **9**: 183–188

Volm M, Hahn EW, Mattern J, Muller T, Vogt-Moykopf I and Weber E (1988b) Five-year follow-up study of independent clinical and flow cytometric prognostic factors for the survival of patients with non-small-cell lung carcinoma. *Cancer Res* **48**: 2923–2928

© 2001 Cancer Research Campaign

Wolley RC, Schreiber K, Koss LG, Karas M and Sherman A (1982) DNA distribution in human colon carcinomas and its relationship to clinical behavior. *J Nat Cancer Inst* **69**: 15–22

Yamaoka N, Uchiyama Y, Kimino K, Akamine S, Matsuo S and Tsuji K (1989) The prognostic significance of nuclear DNA content in non-small-cell lung carcinoma. *Nippon Geka Gakkai Zasshi* **91**: 1608–1616

Yu J, Shaeffer J, Zhu A, Kuban DA, EI-Mahdi AM and Philput CB (1993) Flow cytometric DNA content and clinical outcome in patients with non-small-cell lung cancer given postoperative radiation therapy. *Cytometry* **14**: 428–432

Yusuf S, Cellins R, Peto R, Furberg C, Stampfer MJ, Goldhaber SZ and Hennekens CH (1985) Intravenous and intracoronary fibrinolytic therapy in acute myocardial infarction: overview of results in mortality, reinfarction and side effects for 33 randomised clinical trials. *Eur Heart J* **6**: 556–585

Zimmerman PV, Bint MH, Hawson GAT and Parsons PG (1987) Ploidy as a prognostic determinant in surgically treated lung cancer. *Lancet* **2**: 530–533

*© 2001 Cancer Research Campaign*
